# Safety, tolerability, and outcomes of losartan use in patients hospitalized with SARS-CoV-2 infection: A feasibility study

**DOI:** 10.1371/journal.pone.0244708

**Published:** 2020-12-30

**Authors:** Olena Bolotova, Jeanwoo Yoo, Imran Chaudhri, Luis A. Marcos, Haseena Sahib, Farrukh M. Koraishy, Hal Skopicki, Sahar Ahmad, Sandeep K. Mallipattu

**Affiliations:** 1 Department of Medicine, Stony Brook University, Stony Brook, NY, United States of America; 2 Renal Section, Northport VA Medical Center, Northport, NY, United States of America; Escola Paulista de Medicina, BRAZIL

## Abstract

**Background:**

Retrospective studies on the use of Renin-Angiotensin-Aldosterone System blockade in patients with Coronavirus Disease 2019 (COVID-19) have been informative but conflicting, and prospective studies are required to demonstrate the safety, tolerability, and outcomes of initiating these agents in hospitalized patients with COVID-19 and hypertension.

**Methods and findings:**

This is a single center feasibility study encompassing two cohorts: (1) prospective cohort (April 21, 2020 to May 29, 2020) and (2) retrospective cohort (March 7, 2020 to April 1, 2020) of hospitalized patients with real-time polymerase chain reaction (PCR) positive SARS-CoV-2 by nasopharyngeal swab. Key inclusion criteria include BP > 130/80 and a requirement of supplemental oxygen with FiO_2_ of 25% or higher to maintain SpO_2_ > 92%. Key exclusion criteria included hyperkalemia and acute kidney injury (AKI) at the time of enrollment. Prospective cohort consisted of *de novo* initiation of losartan and continuation for a minimum of 7 days and assessed for adverse events (AKI, hyperkalemia, transaminitis, hypotension) and clinical outcomes (change in SpO_2_/FiO_2_ and inflammatory markers, need for ICU admission and mechanical ventilation). Retrospective cohort consisted of continuation of losartan (prior-to-hospitalization) and assessment of similar outcomes. In the prospective cohort, a total of 250 hospitalized patients were screened and inclusion/exclusion criteria were met in 16/250 patients and in the retrospective cohort, a total of 317 hospitalized patients were screened and inclusion/exclusion criteria were met in 14/317 patients. Most common adverse event was hypotension, leading to discontinuation in 3/16 (19%) and 2/14 (14%) patients in the prospective and retrospective cohort. No patients developed AKI in the prospective cohort as compared to 1/14 (7%) patients in the retrospective cohort, requiring discontinuation of losartan. Hyperkalemia occurred in 1/16 (6%) and 0/14 patients in the prospective and retrospective cohorts, respectively. In the prospective cohort, 3/16 (19%) and 2/16 (13%) patients required ICU admission and mechanical ventilation. In comparison, 3/14 (21%) required ICU admission and mechanical ventilation in the retrospective cohort. A majority of patients in both cohorts (14/16 (88%) and 13/14 (93%) patients from the prospective and retrospective cohort) were discharged alive from the hospital. A total of 9/16 (prospective) and 5/14 (retrospective) patients completed a minimum 7 days of losartan. In these 9 patients in the prospective cohort, a significant improvement in SpO_2_/FiO_2_ ratio was observed from day 1 to 7. No significant changes in inflammatory markers (initiation, peak, and day 7) were observed in either cohort.

**Conclusion:**

In this pilot study we demonstrate that losartan was well-tolerated among hospitalized patients with COVID-19 and hypertension. We also demonstrate the feasibility of patient recruitment and the appropriate parameters to assess the outcomes and safety of losartan initiation or continuation, which provides a framework for future randomized clinical trials.

## Introduction

To date, there are more than 20 million infected individuals with severe acute respiratory syndrome coronavirus 2 (SARS-CoV-2), with more than 750,000 global deaths [1]. Hypertension is one of the most common comorbidities in individuals that progress to Coronavirus Disease 2019 (COVID-19) and is associated with worse prognosis [2–4]. In COVID-19, SARS-CoV-2 uses Angiotensin Converting Enzyme 2 (ACE2) as a receptor to gain viral entry into the host cell [5]. In addition, the use of ACE inhibitors (ACEi)/angiotensin receptor blockers (ARB) has been associated with increased expression of ACE2 receptor in some animal models [6], and, as such, was initially postulated to enhance viral entry into the host cell. However, cross-sectional human studies did not demonstrate increased circulating levels of ACE2 in patients in patients taking RAAS inhibitors [7, 8]. Interestingly, it is postulated that binding of SARS-CoV-2 to ACE2 receptor enhances Angiotensin II (Ang II)-mediated activation of Angiotensin II Type 1 receptor (AT1R), leading to increased inflammation and fibrosis [9]. ACE2 has been reported to counters this high AngII-AT1R-induced pro-inflammatory state by generation of Ang 1–9, 1–7, with Ang 1–7 acting on the Mas receptor to reduce inflammation, vasoconstriction, and pro-fibrotic signaling [6, 10]. In addition, neutral endopeptidases participate in the conversion of Ang I to Ang 1–7 [11] and, as such, RASS blockade might contribute to formation of Ang I-7 via the action of neutral endopeptidase, thereby increasing the salutary effects of Ang 1–7. More recent studies in rodent models also demonstrate that these anti-inflammatory, anti-fibrotic, and vasodilatory effects are, in part, mediated via Ang 1–9 activation of the Angiotensin II Type 2 receptor (AT2R) [12, 13].

While recent retrospective studies have shown that the use of Renin-Angiotensin-Aldosterone System (RAAS) inhibitors might not be associated with a higher likelihood of SARS-CoV-2 infection [14–17], these data have demonstrated conflicting results on the effect of ACEi/ARBs on the course of COVID-19. For instance, Mehta et al., 2020, demonstrated that the use of ACEi/ARBs was associated with increased hospital admission and ACEi were associated with an increase in admission to the intensive care unit (ICU) after propensity-score-weighted analysis [16], while other studies showed no significant differences in mortality and severity of disease among patients taking RAAS inhibitors [18]. In addition, initial studies from China showed that continuation of ACEi/ARBs during hospitalization was associated with lower mortality [18], and in a single-center cohort of hospitalized patients with COVID-19, we recently demonstrated a decreased likelihood of ICU admission and inflammatory burden in patients that were continued on these agents [19]. While majority of these studies suggest that ACEi/ARBs might not need to be discontinued in patients infected with SARS-CoV-2, smaller sample sizes, potential confounders, and retrospective nature of these studies diminishes their potential impact on clinical practice. Furthermore, the impact of *de novo* initiation of RAAS blockade in COVID-19 patients with hypertension remains unexplored. As such recently registered randomized clinical trials (RCTs) have been initiated to investigate the efficacy of RAAS blockade in COVID-19 (NCT04312009, NCT04366050). While *de novo* initiation of RAAS blockade might be beneficial with COVID-19 patients with hypertension, challenges remain clinically in initiating in acutely ill hospitalized patients with hypertension and COVID-19. Here, in this pilot study we demonstrate the feasibility of recruitment, safety, tolerability, and outcomes in initiating losartan *de novo* in hospitalized patients with hypertension and COVID-19. In this study, we selected the use of an ARB (i.e. losartan) due to selective inhibition AT1R as compared to ACEi, which potentially leads to a more upstream inhibition in the RAAS system by targeting ACE and might inhibit the anti-inflammatory effects of Mas receptor activation in COVID-19 [10]. In addition, we show a comparison to a retrospective cohort of patients with COVID-19 that were continued on losartan during their hospitalization.

## Methods

### Study design and participants

This is a single-center study encompassing two cohorts: (1) prospective cohort and (2) retrospective cohort of hospitalized patients with real-time PCR positive SARS-CoV-2 by nasopharyngeal swab. All patients included in the study cohort reached the final outcome of either discharged alive from the hospital or death. This study was approved by the Stony Brook University Institutional Review Board.

The prospective cohort included hospitalized patients with COVID-19 at Stony Brook University Hospital from April 21, 2020 to May 29, 2020, inclusive of these dates. A complete list of inclusion and exclusion criteria, with the respective definitions, is provided in the S1 File. Written informed consent was obtained by the study team for patients who met inclusion and exclusion criteria and were started on losartan 25 mg daily. The dose of losartan was increased incrementally by 25 mg daily up to 100 mg daily (as tolerated) until the goal BP of < 130/80 was met. Assessment of vital signs, clinical symptoms, and laboratory values was performed daily while the patients were on losartan during hospitalization. Patients were also monitored for adverse events daily during hospitalization after initiation of losartan. In patients that survived the hospitalization, post-discharge follow-up was conducted 28 days after discharge from the hospital.

The retrospective cohort included hospitalized patients with COVID-19 with a history of losartan use prior to admission at Stony Brook University Hospital from March 7, 2020 to April 1, 2020, inclusive of these dates. Inclusion and exclusion criteria were as in the prospective cohort, with the exception of exclusion criteria of active use of RAAS blockade. Assessment of daily parameters were conducted as in the prospective cohort. In patients that survived the hospitalization, written informed consent was obtained by the study team to conduct post-discharge follow-up at 90 days after discharge from the hospital.

### Data collection and definition of variables

Vitals signs and laboratory values were collected through the electronic health record in both cohorts and post-discharge follow-up was conducted via phone call to the patient or legally authorized representative. Vital signs included blood pressure with daily average systolic blood pressure (SBP), diastolic blood pressure (DBP) and ratio of SpO_2_ to FiO_2_ (S/F). Monitoring of clinical symptoms included cough, fever, and dyspnea. Collected laboratory values consisted of serum creatinine (sCr), potassium (K^+^), alanine aminotransaminase (ALT), aspartate aminotransferase (AST), D-dimer, lactate dehydrogenase (LDH), ferritin, c-reactive protein (CRP), erythrocyte sedimentation rate (ESR). All data was collected daily while the patients were on losartan from the time of losartan initiation.

Clinical outcomes included the need for ICU admission, respiratory failure requiring mechanical ventilation (MV), length of hospital stay, death, 28-day and 90-day mortality and readmission.

Reported adverse events include AKI, hyperkalemia, hypotension and transaminitis. AKI was defined as a rise in sCr of 0.3 mg/dl within any 48-hour time period during the hospitalization or ≥ 1.5 times increase from admission sCr based on KDIGO guidelines [20]. Transaminitis was defined as a sustained increase in AST and/or ALT greater than three times the upper limit of normal. Hypotension was defined as average BP < 110/60 mmHg measured as an average of three BP values measured 8 hours apart. Hyperkalemia was defined as K^+^ > 5.5 mEq/L.

In patients that completed a minimum of 7 days of losartan during their hospitalization, we assessed for changes in S/F ratio and inflammatory markers. A composite inflammatory score (0–15) was calculated based on combined quartile average data that included Ferritin, LDH, ESR, D-Dimer, CRP from the time of losartan initiation to the “peak” value of these markers as recently described [21]. Patients that did not have values, were assigned 0 points. “Peak” values for these inflammatory markers were also reported independently.

### Statistical analysis

Continuous variables were presented as Mean ± SD and categorical variables presented as numbers and percentages. P-values were calculated with sample paired t-test for the values within one cohort, and Welch’s t-test for continuous variables between two cohorts, and Fisher’s exact test was used to calculate p-values for categorical variables. Results were considered to be statistically significant if a p-value was < 0.05. These calculations were performed with SPSS and Prism 8 software. A simple linear regression was used to analyze the change in S/F ratio in each individual patient and a combined S/F ratio linear regression for all patients who completed a minimum of 7 days of losartan during hospitalization.

## Results

### Baseline characteristics of the study patients

**Fig 1:**
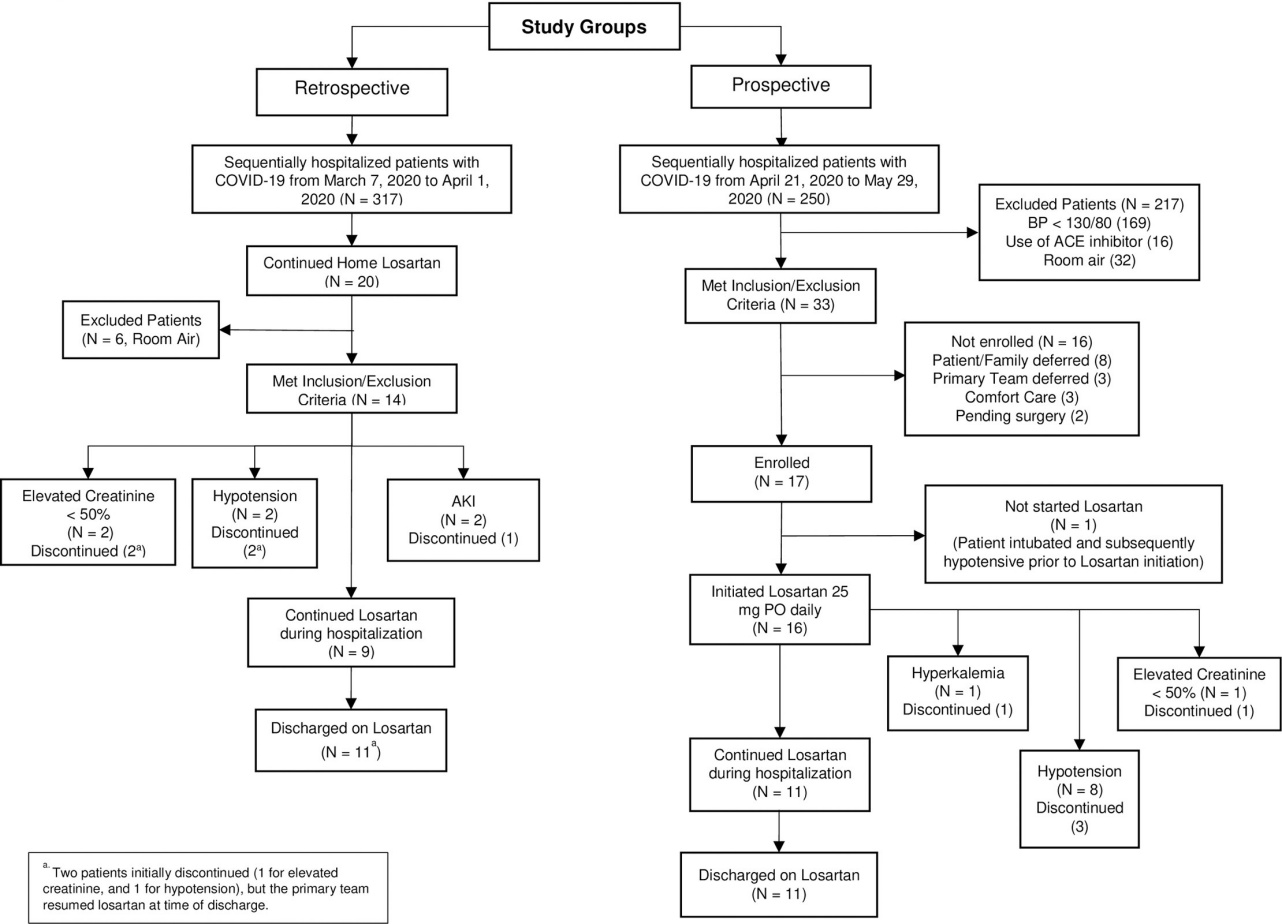
Flow chart of selection of the two study cohorts in the study, prospective and retrospective cohort. Total number of individuals that met the specific inclusion/exclusion criteria for enrollment as well as adverse events are shown.

In the prospective arm of the study, a total of 250 sequentially hospitalized patients with PCR positive SARS-CoV-2 were screened from April 21, 2020 to May 29, 2020 (inclusive of these dates), and 33 met the inclusion/exclusion criteria for enrollment. However, only 16/250 were ultimately enrolled in the prospective arm of the study as provided in **Fig 1.** In the retrospective arm of the study, a total of 317 sequentially hospitalized patients with PCR positive SARS-CoV-2 from March 7, 2020 to April 1, 2020 (inclusive of these dates) were screened, and 14/317 patients met the inclusion/exclusion criteria for enrollment **(Fig 1).**

The mean age of the participants was similar in both cohorts, 62 ± 14 and 66 ± 12 years in the prospective and retrospective cohorts, respectively. The majority of patients in the both groups were male, 13 (81%) in the prospective arm and 11 (79%) in the retrospective arm. All patients in both cohorts had a body mass index (BMI) > 25, with an average BMI of 33 ± 10 and 30 ± 4 in the prospective and retrospective cohort, respectively **(Table 1).**

**Table 1:** Presenting characteristics in the study cohort.

	Prospective	Retrospective	*P-value*
	**N = 16**	**N = 14**	
**Demographics**			
Age, y	62 ± 14	66 ± 12	0.50
Male	13 (81%)	11 (79%)	1.00
BMI	33 ± 10	30 ± 4	0.27
**Comorbidities**			
Charlson Comorbidity Index	3.5 ± 2.8	3.9 ± 2.7	0.67
Hypertension	11 (69%)	14 (100%)	0.05
Diabetes	4 (25%)	6 (43%)	0.44
Coronary Artery Disease	2 (13%)	3 (21%)	0.64
Vascular Disease	1 (6%)	1 (7%)	1.00
Heart Failure with preserved EF	2 (13%)	2 (14%)	1.00
Chronic Kidney Disease	1 (6%)	1 (7%)	1.00
End Stage Kidney Disease	0 (0%)	1 (7%)	0.47
Chronic Obstructive Pulmonary Disease	3 (19%)	2 (14%)	1.00
Other Lung Disease	3 (19%)	2 (14%)	1.00
**Blood Pressure on Admission**			
SBP, mmHg	145 ± 22	130 ± 22	0.08
DBP, mmHg	82 ± 12	74 ± 13	0.12
MAP, mmHg	101 ± 14	91 ± 14	0.09
**Kidney Function on Admission**			
BUN, mg/dL	23 ± 22	18 ± 5	0.39
sCr, mg/dL	1.0 ± 0.6	1.1 ± 1.9	0.47
eGFR, ml/min/1.73m^2^	84 ± 31	67 ± 21	0.09
**S/F Ratio on Admission**	273 ± 117	309 ± 92	0.35

Data are presented as a number and percentage, mean ± standard deviation (SD). P-value < 0.05 considered statistically significant in analysis.

BMI, body mass index; ACEi, angiotensin converting enzyme inhibitor; ARB, angiotensin receptor blocker; SBP, systolic blood pressure; DBP, diastolic blood pressure; MAP, mean arterial pressure; BUN, blood urea nitrogen; sCr, serum creatinine; eGFR, estimated glomerular filtration rate by CKD-EPI equation; S/F, SO2/FiO2.

In comparison to the retrospective cohort, not all patients in the prospective cohort had a previous documented history of hypertension prior to admission **(Table 1)**. Charlson comorbidity index was on average greater than 3 in both cohorts, 3.5 ± 2.8 and 3.9 ± 2.7 in the prospective and retrospective cohort, respectively, suggesting significant burden of disease in both cohorts. Average SBP was higher in the prospective cohort (145 ± 22 mmHg) as compared to the retrospective cohort (130 ± 22 mmHg), but was not statistically significant, p = 0.08. There was no difference in S/F ratio and kidney function on enrollment between two cohorts **(Table 1)**.

### Losartan dose, time of initiation, and duration in the study cohort

While patients that were actively using ACEi were excluded from prospective arm of the study, 2/16 patients from prospective group were on ACEi and 2/16 patients were on ARB prior to admission and were started on losartan after a minimum washout period of 4 days.

Not surprisingly, patients in the prospective cohort trended towards a longer lag time for initiation of losartan (7.8 ± 11.0 days) as compared to the retrospective cohort (3.5 ± 7.1 days), but this did not reach statistical significance (p = 0.21). While the average daily dose of losartan was significantly higher in the retrospective cohort as compared to the prospective cohort (65 ± 29 mg versus 32 ± 16 mg, p = 0.001), the cumulative dose during hospitalization between both groups were similar **(Table 2).** These findings suggest that a lower daily dose of losartan was tolerated longer in the prospective cohort as compared to the retrospective cohort, but this did not reach statistical significance (8.9 ± 7.5 versus 5.5 ± 4.4 days, p = 0.13). Patients in the retrospective cohort were also discharged on a higher daily dose of losartan as compared to the patients in the prospective cohort (66 ± 28 mg vs. 28 ± 11 mg, p = 0.002). Finally, 11/16 (69%) patients from the prospective cohort were discharged on losartan as compared to 11/14 (79%) in the retrospective cohort, suggesting that a majority of patients in both cohorts tolerated the use of losartan during the hospitalization **(Table 2).**

**Table 2:** Dosing of losartan in the study cohorts.

	Prospective	Retrospective	*P-value*
	N = 16	N = 14	
**History of ACEi/ARB Use**			
ACEi only	2 (13%)	0	0.49
ARB only	2 (13%)	14 (100%)	<0.0001
Total	4 (25%)	14 (100%)	<0.0001
**In Hospital Losartan**			
Time to Losartan Initiation, days	7.8 ± 11.0	3.5 ± 7.1	0.21
Avg Duration, days	8.9 ± 7.5	5.5 ± 4.4	0.13
Avg Total Dose, mg	309 ± 338	291 ± 221	0.86
Avg Daily Dose, mg	32 ± 16	65 ± 29	0.001
**Discharge Losartan**			
Discharged Alive on Losartan	11 (69%)	11 (79%)	0.69
Avg Dose on Discharge, mg	28 ± 11	68 ± 32	0.002

Data is presented as number and percentage, mean ± standard deviation (SD). P-value < 0.05 considered statistically significant in analysis.

ACEi, angiotensin converting enzyme inhibitor; ARB, angiotensin receptor blocker.

### Safety of losartan use in the study cohorts

To assess the safety of losartan use in both study cohorts, we assessed the incidence of hypotension, transaminitis, AKI, and hyperkalemia **(Tables 3 and 4).** While hypotensive episodes were noted in 8/16 (50%) of the patients in the prospective cohort after initiation of losartan, only 3 (19%) led to discontinuation of losartan. In these three patients, one required an intravenous bolus of fluids and initiation of vasopressor support, one required a bolus of fluids to maintain SBP > 100 mmHg (but was eventually restarted on losartan at discharge), and losartan was discontinued in the third patient due to holding parameters of SBP < 120 mmHg by the primary team. Similarly, discontinuation of losartan due to persistent hypotension only occurred in 2 (14%) patients in the retrospective cohort **(Table 3).**

**Table 3:** Adverse events in the study cohort.

	Prospective	Retrospective	*P-value*
	N = 16	N = 14	
**Adverse Events leading to discontinuation of Losartan**			
Hypotension^a^	3 (19%)^b^	2 (14%)^c^	1.00
AKI^d^	0 (0%)	1 (7%)	0.47
Hyperkalemia^e^	1 (6%)	0 (0%)	1.00
**Total Adverse Events**			
Hypotension	8 (50%)	3 (21%)	0.14
Percentage (%) of days of hypotension per total duration of Losartan^f^	9 ± 11	4 ± 13	0.26
AKI	0 (0%)	2 (14%)^g^	0.21
Hyperkalemia	1 (6%)	0 (0%)	1.00
Transaminitis^h^	2 (13%)	2 (14%)	1.00

Data is presented as Number and percentage, Mean ± Standard Deviation. P-value < 0.05 considered statistically significant in analysis.

^a.^ Hypotension, defined by average SBP/DBP < 110/70 over 3 readings 8 hours apart on one day.

^b.^ Hypotension in prospective cohort requiring discontinuation of losartan, one patient with BP of 79/51 mmHg and Atrial fibrillation with heart rate to ~160 beats/min (8 days post-losartan), second patient with BP of 86/49 mmHg (3 days post-losartan), subsequently resumed on losartan at discharge, third patient with BP of 103/61 mmHg (2 days post-losartan) in setting of urinary tract infection.

^c.^ Hypotension in retrospective cohort requiring discontinuation of losartan, one patient with BP of 91/59 mmHg after intubation (6 days post-losartan), second patient with hypotension to 100/61 mmHg after intubation (1-day post-losartan), subsequently resumed losartan on discharge.

^d.^ AKI, Acute kidney injury, defined according to KDIGO guidelines.

^e.^ Hyperkalemia defined as serum potassium ≥ 5.5 mEq/L.

^f.^ Percentage days of hypotension of total days on losartan averaged for all patients in each cohort.

^g.^ Retrospective cohort with 2 patients with AKI, one patient discontinued losartan, second patient continued losartan and AKI resolved.

^h.^ Transaminitis defined as elevation of transaminase ≥ 3X upper limit of normal (ALT ≥ 123, AST ≥ 120).

**Table 4:** Clinical outcomes & disposition after initiation of losartan in the study cohort.

	Prospective	Retrospective	*P-value*
	N = 16	N = 14	
**BP**^**a**^			
Avg Daily SBP, mm Hg	130 ± 12	139 ± 21	0.16
Avg Daily DBP, mm Hg	72 ± 6	76 ± 6	0.21
**Transaminases**^**a**^			
Avg Daily ALT, IU/L	43 ± 39	53 ± 38	0.50
Avg at Peak ALT, IU/L^b^	59 ± 52	61 ± 50	0.96
*P-value*	0.32	0.66	
Avg Daily AST, IU/L	30 ± 20	52 ± 30	0.05
Avg at Peak AST, IU/L^b^	40 ± 24	60 ± 41	0.15
*P-value*	0.22	0.58	
**sCr**^**a**^			
Avg Daily, mg/dL	0.8 ± 0.5	0.9 ± 0.3	0.38
Avg at Peak, mg/dL^b^	0.9 ± 0.5	1.0 ± 0.3	0.59
*P-value*	0.58	0.94	
**Outcomes after initiation of Losartan**			
ICU Admission	3 (19%)	3 (21%)	1.00
Invasive Mechanical Ventilation	2 (13%)	3 (21%)	0.64
Patient Disposition			
Discharged Alive	14 (88%)	13 (93%)	1.00
Death	2 (13%)	1 (7%)	1.00

Data is presented as number and percentage, mean ± standard deviation (SD). P-value<0.05 considered statistically significant in analysis.

^a.^ Values obtained from the time of initiation of Losartan until discontinuation or discharge.

^b.^ Peak values while on Losartan.

Transaminitis developed in 2/16 (13%) and 2/14 (14%) patients developed transaminitis in prospective and retrospective cohorts, respectively, however the transaminitis was not persistently elevated to require discontinuation in either. None of the patients in the prospective cohort developed AKI, but 1/14 (7%) patients in the retrospective cohort developed AKI that led to discontinuation of losartan. Collectively, these data suggest that the use of losartan in hospitalized hypertensive patients with COVID-19 was well-tolerated, with hypotension being the most common reason for discontinuation of the agent.

### Outcomes with the use of losartan in the study cohort

A majority of patients in both study cohorts did not require MV or admission to the ICU **(Table 4).** Furthermore, 14/16 (88%) and 13/14 (93%) of patients in the prospective and retrospective cohorts were discharged alive after initiation of losartan **(Table 4).** However, it is noteworthy that only 9/16 (56%) and 5/14 (36%) in the prospective and retrospective cohorts completed a minimum of 7 days of losartan during hospitalization. Reasons include the duration of hospitalization less than 7 days, late initiation of the losartan during hospitalization, and discontinuation of losartan due to adverse events.

To evaluate the effects of losartan in patients that received a minimum 7 days of losartan, we measured the change in markers of inflammation (time of initiation to peak values) and change in S/F ratio from the onset of losartan use. We observed no significant increases in individual markers of inflammation (Ferritin, LDH, D-dimer, CRP) as well as the change in the composite inflammatory score from day 1 to 7 **(Table 5)**, with the exception of the increase in ESR in the prospective cohort (44 ± 16 vs. 69 ± 27 IU/L, p = 0.03).

**Table 5:** Inflammatory markers in the study cohort.

	Prospective	Retrospective	*P-value*
	N = 9	N = 5	
**Day 1 Composite**^**a**^	6.8 ± 4.9	3.4 ± 3.4	0.16
**Day 7 Composite**^**a**^	6.3 ± 3.9	2.4 ± 3.4	0.08
*P-value* (Day 1 vs Day 7)	0.83	0.65	
**Peak Composite**^**a**^	7.2 ± 3.6	4.8 ± 3.4	0.24
*P-value* (Day 1 vs Peak)	0.83	0.53	
**Ferritin**, ng/mL			
Initiation	748 ± 643	1192 ± 638	0.49
Peak^b^	817 ± 619	1604 ± 211	0.01
*P-value*	0.82	0.52	
**LDH**, IU/L			
Initiation	370 ± 135	254 ± 32	0.06
Peak^b^	456 ± 157	318 ± 159	0.27
*P-value*	0.25	0.56	
**ESR**, IU/L			
Initiation	44 ± 16	55 ± 28	0.58
Peak^b^	69 ± 27	86 ± 40	0.45
*P-value*	0.03	0.25	
**D-dimer**, ng/mL			
Initiation	853 ± 700	797^c^	-
Peak^b^	942 ± 640	1539 ± 978	0.54
*P-value*	0.79	-	
**CRP**, mg/dL			
Initiation	4.5 ± 4.8	3.9 ± 3.5	0.81
Peak^b^	8.2 ± 8.2	7.7 ± 6.2	0.89
*P-value*	0.26	0.31	

Data is presented as mean values, including Composite, Initiation, and Peak values (Mean and Standard deviation) in patients that received a minimum of 7 days of losartan.

LDH, lactate dehydrogenase; ESR, erythrocyte sedimentation rate; CRP, C-reactive protein

P<0.05 considered statistically significant in analysis.

^a.^ Composite inflammatory values range from 0–15 based on combined quartile data including Ferritin, LDH, ESR, D-Dimer, CRP values.

^b.^ Peak inflammatory values while on Losartan in patients who received minimum 7 days of Losartan.

^c.^ D-dimer level available in only one patient in the retrospective group.

**Fig 2:**
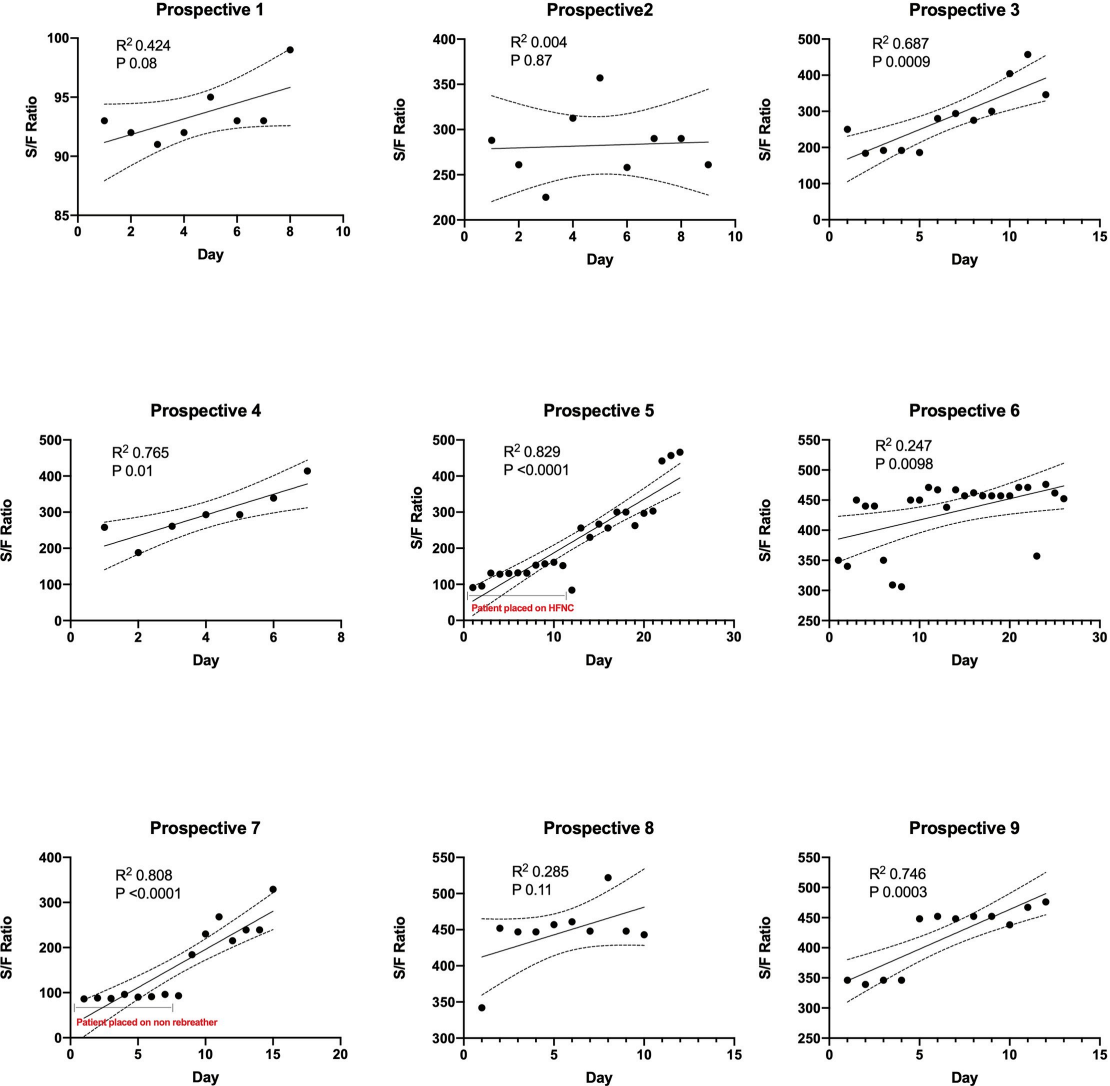
Linear regression of S/F ratio for each patient that completed a minimum of 7 days of losartan in the prospective cohort from the start of losartan during hospitalization until the time of discharge or discontinuation of losartan. HFNC, High Flow Nasal Cannula. Data expressed as daily S/F ratio, with best fit line (R^2^, slope with 95% confidence interval, P-value for statistically significant non-zero slope).

**Fig 3:**
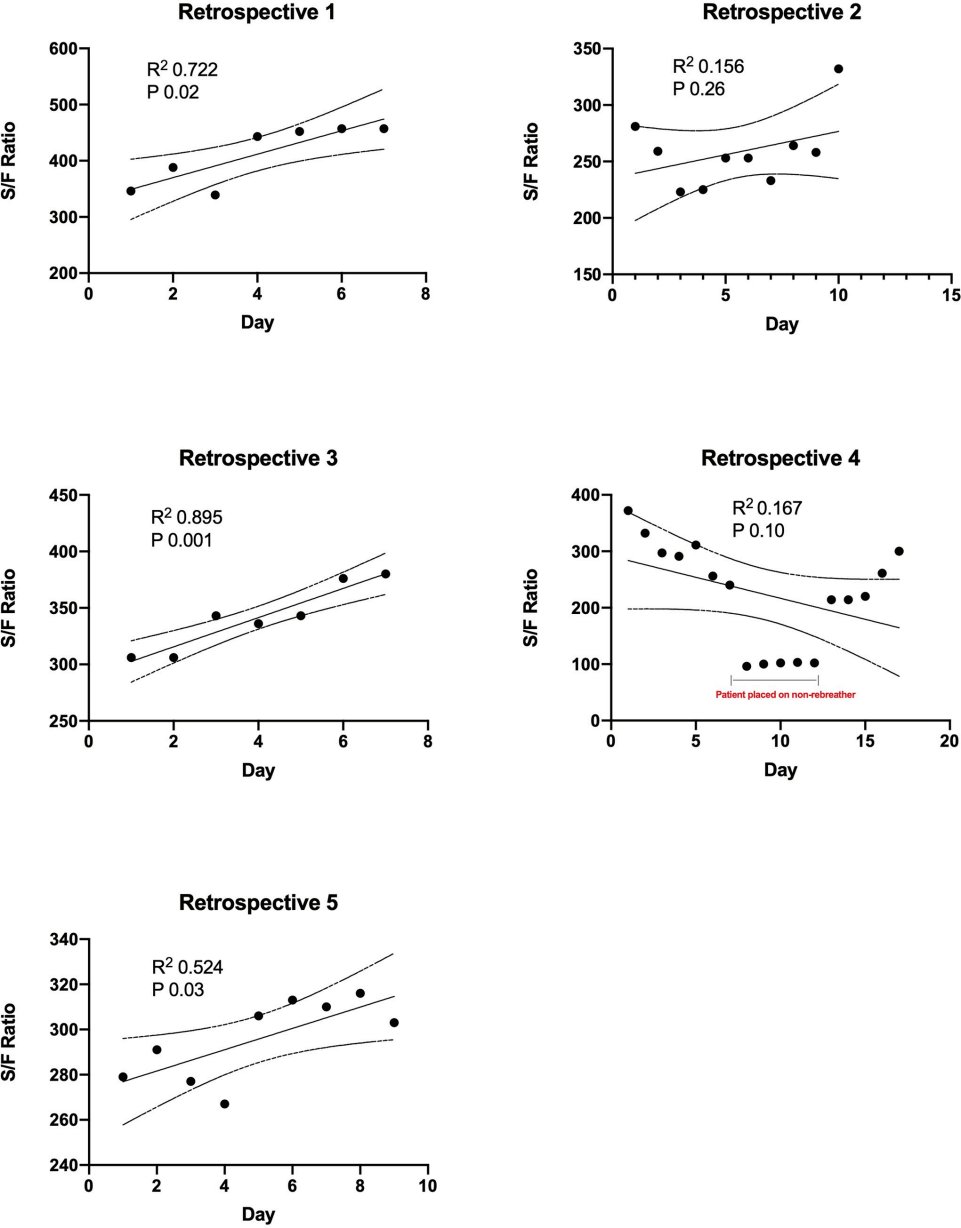
Linear regression of S/F ratio for each patient that completed a minimum of 7 days of losartan in the retrospective cohort from the start of losartan during hospitalization until the time of discharge or discontinuation of losartan. Data expressed as daily S/F ratio, with best fit line (R^2^, slope with 95% confidence interval, P-value for statistically significant non-zero slope).

**Fig 4:**
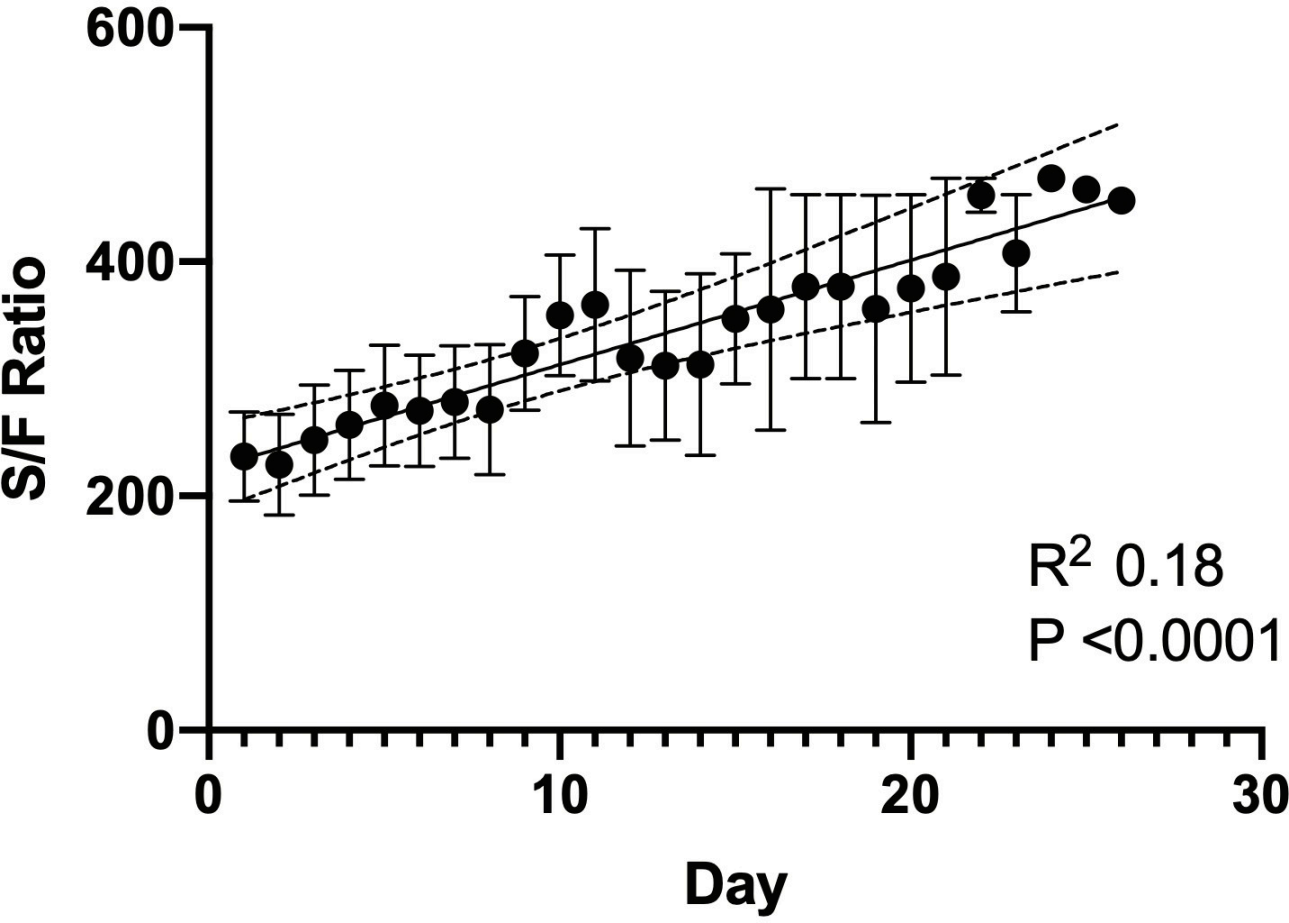
Linear regression of S/F ratio for all patients that completed a minimum of 7 days of losartan in the prospective cohort from the start of losartan during hospitalization until the time of discharge or discontinuation of losartan. Data expressed as mean ± SEM of S/F ratio for each day, with best fit line (R^2^, slope with 95% confidence interval, P-value for statistically significant non-zero slope).

Linear regression was utilized to evaluate the change in S/F ratio for patients who completed a minimum of 7 days of losartan in both cohorts on an individual basis **(Figs 2 and 3).** In all patients in both cohorts, the S/F ratio remained unchanged or improved from the onset of losartan initiation to the end of the hospitalization period, with a statistically significant improvement in 6/9 patients by day 7 after initiating losartan in the prospective cohort. In patients that initially had no improvement in S/F ratio, there was an eventual improvement after day 7 of losartan use. In the retrospective cohort, 3/5 patients showed a significant improvement by day 7 after losartan initiation. Collectively, we also observed an improvement in S/F ratio during hospitalization in the prospective cohort (r^2^ 0.18, p = 0.0001) **(Fig 4).** Finally, patients that did not complete a minimum of 7 days of losartan showed no significant changes in S/F ratio in the prospective cohort (r^2^ 0.009, p = 0.7).

**Fig 5:**
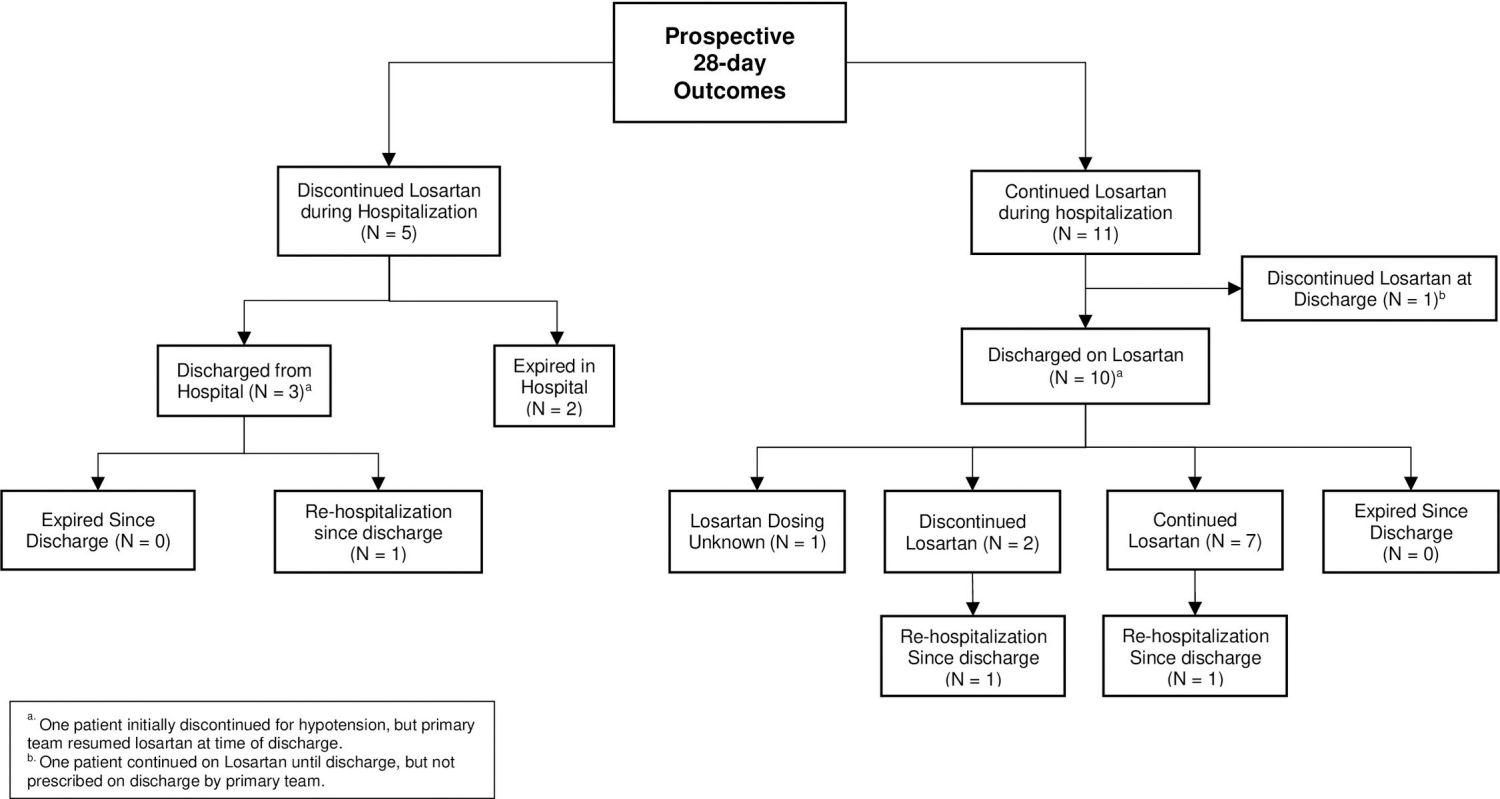
Flow chart for outcomes for patients at 28 days post-discharge in the prospective cohort.

To evaluate the effects of losartan after discharge from the hospital, we conducted post-discharge follow-up at day 28 in the prospective cohort and day 90 in the retrospective cohort **(Fig 5, Tables 6 and 7)**. In patients who completed a minimum of 7 days of losartan during hospitalization in the prospective cohort, no patients died at the end of 28 days, but 2/11 required re-hospitalization and 5/11 remained dependent on supplemental oxygen therapy. It is noteworthy that only 7/11 remained on losartan at 28 days post-discharge **(Table 6).** Due to the retrospective nature of the cohort, only 90-day outcomes were collected for these patients and, as such, data was only available for 8/14 patients in the cohort **(Table 7)**. Similar to the prospective cohort, no patients died during the post-discharge follow-up and only 1/8 patients remained on supplemental oxygen and 1/8 patients required re-hospitalization after discharge.

**Table 6:** Post-discharge outcomes in prospective cohort at 28 days.

Total	N = 16
**Discontinued Losartan during Hospitalization**^**a**^	**N = 5**
** 28-day outcomes**	
Expired in hospital	2 (40%)
Discharged from hospital	3 (60%)
Resumed Losartan at discharge^b^	1 (20%)
Re-Hospitalization since discharge	1 (20%)
Expired since discharge	0
**Continued Losartan during Hospitalization**^**c**^	**N = 11**
** 28-day outcomes**	
Expired in hospital	0
Discharged from hospital	11 (100%)
Taking Losartan	7 (64%)
Losartan Dose, mg	29 ± 9
Discontinued Losartan at discharge^d^	1 (9%)
Discontinued Losartan since discharge	2 (18%)
Follow-up Dosing unavailable^e^	1 (9%)
Requiring Oxygen	5 (45%)
Re-Hospitalization since discharge	2 (18%)
Expired since discharge	0

Data is presented as number and percentage, Mean ± Standard Deviation.

^a.^ Patients who received losartan inpatient but discontinued prior to death or discharge.

^b.^ Losartan was held in one patient during hospitalization but resumed 2 days prior to discharge, and prescribed at discharge.

^c.^ Patients who received losartan inpatient and continued until discharge.

^d.^ One patient continued losartan during hospitalization but discontinued at discharge.

^e.^ Unable to reach patient to confirm Losartan dosing at 28 days post-discharge.

**Table 7:** Post-discharge outcomes in retrospective cohort at 90 days.

Total	N = 14
Data unavailable post-discharge	N = 6
** 90-day outcomes**	**N = 8**
On Losartan	8 (100%)
Avg Losartan Dose, mg	75 ± 25 mg
Re-Hospitalization since discharge	1 (13%)
Requiring Oxygen	1 (12%)
Expired since discharge	0 (0%)

Data is presented as Number and percentage, Mean ± Standard Deviation.

Percentages shown for patients with available data 90 days post-discharge.

## Discussion

In this pilot study, we demonstrate the safety, tolerability, and outcomes of losartan use in hospitalized patients with COVID-19 and hypertension. We also highlight the feasibility of recruiting patients based on our inclusion and exclusion criteria. In addition, we show a comparison of outcomes of patients that were continued on losartan during hospitalization. Key observations include that the use of losartan is relatively well-tolerated and the primary reason for discontinuation is sustained hypotensive episodes. Furthermore, starting at a dose of 25 mg of losartan might have allowed for prolonged use during hospitalization. Initiation of losartan during hospitalization also did not result in worsening of inflammatory markers or oxygenation status in patients that completed a minimum of 7 days of losartan. There were suggestions of lung protection with losartan use, including the observation of an improvement in S/F ratio during hospitalization in the prospective cohort, however these findings would need validation through a properly designed randomized clinical trial (RCT). Our findings can serve to guide target outcomes measures for such trials.

The most common adverse event associated with losartan use that required discontinuation was persistent hypotension (requiring intravenous fluid bolus and initiation of vasopressor support). While not statistically significant, a trend towards a higher rate of hypotensive episodes in the prospective cohort as compared to the retrospective cohort might be explained by the *de novo* initiation of losartan in the prospective cohort as well as the long-standing history of hypertension in the retrospective cohort. In addition, we observed that five out of the eight patients from the prospective group were able to tolerate losartan despite intermittent episodes of BP < 110/70 mmHg. As such, a better definition of hypotension is required for the future RCTs, such as sustained BP < 100/60 mmHg that requires discontinuation of losartan and/or requiring intravenous fluid bolus or vasopressor support. In addition, an appropriate control group for losartan might also need to include the use of other anti-HTN agents independent of RAAS blockade to test whether the salutary effects of losartan in COVID-19 are independent of improved BP.

Recruitment remained a challenge in the study, with majority of the patients excluded in the prospective cohort due to BP < 130/80 mmHg. Liberalization of the BP criteria in the inclusion criteria might facilitate improved recruitment in future prospective or RCT. While several RCTs are currently underway in testing the potential efficacy of RAAS blockade in COVID-19, (NCT04312009, NCT04366050), similar challenges might be encountered in patient recruitment.

In addition to hard outcomes such as death and need for MV or ICU admission, assessment of the change in S/F ratio (i.e. oxygenation) and inflammatory markers might serve as useful surrogate markers to assess efficacy of losartan in future RCTs. In addition, we demonstrate that post-discharge follow-up remains an important outcome to measure, especially as patients with COVID-19 are being discharged on supplemental oxygen, which might place them at a higher risk for potential readmission.

Several limitations exist in this pilot study. For instance, not all patients had documented S/F ratio and inflammatory markers for all our patients at day 7. In our retrospective cohort, some of Inflammatory markers were missing, which was likely due to early phases of the pandemic when testing for inflammatory markers was not as common. While the focus of this pilot study was to primarily test the safety and tolerability of losartan use and feasibility of patient recruitment, small sample sizes and lack of placebo control remains a limitation. While New York was the initial epicenter during the start of the COVID-19 pandemic in the United States, decreasing hospitalization due to state-wide measures have significantly mitigated the number of patients hospitalized with COVID-19. Nonetheless, we demonstrate the safety, tolerability, and feasibility of administering losartan in hospitalized patients with COVID-19 and hypertension. Future studies will likely require a multi-center experience to improve patient recruitment. Another limitation is that our assessment of oxygenation was limited by the lack of data on arterial PaO_2_, due to the difficulty in acquiring daily arterial blood gas in non-ICU patients. While the S/F ratio is proven in the literature for use in the characterization of oxygenation status, it can overemphasize the true degree of supplemental oxygenation in use. As such, we are confident that the observed trends towards improvement in S/F ratio represent meaningful improvements in oxygenation status. However, these findings need to be validated with future RCTs.

In conclusion, the use of losartan in hospitalized patients with COVID-19 and hypertension was generally well tolerated and did not cause exacerbation of disease. However, feasibility of recruitment remains a challenge based on our current inclusion/exclusion criteria. This feasibility study provides a framework for designing future RCTs with appropriate inclusion/exclusion criteria and parameters to assess the therapeutic efficacy and safety of losartan in COVID-19.

## Supporting information

S1 File Supplementary methods. (DOCX)
