# Supplementary material for: Safety, tolerability, and outcomes of losartan use in patients hospitalized with SARS-CoV-2 infection: A feasibility study
Source: PLoS One. 2020 Dec 30;15(12):e0244708. doi: 10.1371/journal.pone.0244708 (PMC7773257; doi:10.1371/journal.pone.0244708)
Supplement: S1 File — (DOCX) [file pone.0244708.s001.docx]

**SUPPLEMENTARY METHODS**

**Inclusion criteria**

1. Real-time PCR positive SARS-CoV-2 by nasopharyngeal swab.
2. BP > 130/80 mmHg.
3. Requirement of 0.25 or higher FiO_2_ supplementation in order to maintain SpO_2_ > 92%.

**Exclusion criteria**

1. Age < 18 years.
2. Hyperkalemia (Serum potassium > 5.5 meq/L).
3. Acute Kidney Injury (increase of serum creatinine > 50% of the baseline or decrease in urine output < 0.5 ml/kg/hr for ≥ 12 hours).
4. Active use of RAAS blockade agents.
5. Prior intolerance or allergy to ARB.
6. Pregnancy or breast feeding.
7. In females of childbearing age, unwillingness to use birth control for the duration of the study.
8. Inability to obtain informed consent from patient, health care proxy or surrogate decision maker.
